# Sulforaphene Suppresses Adipocyte Differentiation via Induction of Post-Translational Degradation of CCAAT/Enhancer Binding Protein Beta (C/EBPβ)

**DOI:** 10.3390/nu12030758

**Published:** 2020-03-13

**Authors:** Hee Yang, Min Jeong Kang, Gihyun Hur, Tae Kyung Lee, In Sil Park, Sang Gwon Seo, Jae Gak Yu, Yong Sang Song, Jung Han Yoon Park, Ki Won Lee

**Affiliations:** 1 Center for Food and Bioconvergence, Seoul National University, Seoul 08826, Korea; yhee6106@snu.ac.kr; 2 Advanced Institutes of Convergence Technology, Seoul National University, Suwon 443-270, Korea; jyoon@hallym.ac.kr; 3 Department of Agricultural Biotechnology, Seoul National University, Seoul 151-921, Korea; bluesky3031@hanmail.net (M.J.K.); ginnyhur@hanmail.net (G.H.); vluetk@snu.ac.kr (T.K.L.); insil@snu.ac.kr (I.S.P.); seo0414@naver.com (S.G.S.); ayuhanabi4@naver.com (J.G.Y.); yssong@snu.ac.kr (Y.S.S.); 4 Department of Obstetrics and Gynecology, Seoul National University College of Medicine, Seoul 03080, Korea; 5 Research Institute of Agriculture and Life Sciences, Seoul National University, Seoul 151-921, Korea

**Keywords:** adipogenesis, CCAAT/enhancer-binding protein beta, obesity, post-translational degradation, sulforaphene

## Abstract

Adipocyte differentiation (adipogenesis) is a crucial process that determines the total number and size of mature adipocytes that will develop. In this study, the anti-adipogenic effect of sulforaphene (SFEN), a dietary isothiocyanate (ITC) derived from radish, is investigated both in 3T3-L1 pre-adipocytes and in human adipose tissue-derived stem cells. The results revealed that SFEN significantly inhibit adipogenic cocktail-induced adipocyte differentiation and lipid accumulation at the early stage of adipogenesis. Additionally, the effects are more potent compared to those of other ITCs derived from various cruciferous vegetables. As a related molecular mechanism of action, SFEN promotes the post-translational degradation of CCAAT/enhancer-binding protein (C/EBP) β by decreasing the stability of C/EBPβ, which is responsible for decreasing the expression of master regulatory proteins such as peroxisome proliferator-activated receptor γ and C/EBPα. Collectively, these results suggest that the intake of SFEN-enriched natural materials could be helpful as a strategy for preventing obesity.

## 1. Introduction

Obesity is a chronic disease and a major human health challenge worldwide [1]. The global obesity epidemic represents a serious problem in modern society because obesity is correlated with an increased risk of severe chronic diseases such as type 2 diabetes, cardiovascular diseases, and certain types of cancer, including colorectal and breast cancer [2,3]. Obesity arises from the expansion of body fat mass through increasing the abundance and average volume of adipocytes [4,5]. Adipocyte differentiation (adipogenesis) is the process by which adipocyte progenitors such as pre-adipocytes are converted into lipid-laden mature adipocytes. Adipocytes progenitors can undergo increase of the number or differentiation in adipose tissue formation. There are several cell models including 3T3-L1 pre-adipocytes well established as mimic in vitro model for adipogenesis over several days under the control of hormonal stimuli [6,7,8]. Adipogenesis is controlled by a cascade of several transcriptional factors that regulate the expression of genes for mature adipocyte phenotypes [6], including peroxisome proliferator-activated receptor γ (PPARγ) and CCAAT/enhancer-binding protein (C/EBP) α, which are master regulators of adipogenesis [9].

C/EBPβ is an important transcription factor in obesity [10,11]. In the absence of the *C/ebpβ* gene in adipocytes, the induction of PPARγ and C/EBPα is insufficient, and the differentiation of mature adipocytes is limited [12,13,14]. For instance, knocking out the *C/ebpβ* gene protected mice from high-fat diet (HFD)-induced obesity and fatty liver by decreasing body fat mass and serum lipid levels including triglycerides, free fatty acids, and cholesterols, compared to untreated HFD-fed wild-type mice [10]. Moreover, C/EBPβ-deficient mice exhibited decreased inflammation and increased energy expenditure, which were attributed to the up-regulated expression of mitochondrial, browning, and β-oxidation-related genes such as *Prdm16*, *Cidea*, uncoupling protein (*Ucp*)*1*, and *Ucp3*, compared to gene expression in wild-type mice [10,15,16]. Even in mice fed a normal diet, C/EBPβ deficiency reduced fat mass and altered the body composition in both male and female mice [17]. Therefore, attenuating C/EBPβ expression is an attractive target for ameliorating obesity.

Isothiocyanates (ITCs) are natural compounds, which are formed by the enzymatic breakdown of glucosinolates found in cruciferous vegetables such as broccoli, cabbage, and radish [18]. ITCs are characterized by a common N=C=S structure and various side chains, and include sulforaphene (SFEN), sulforaphane (SFN), iberin (IBR), erucin (ERC), allyl isothiocyanate (AITC), benzyl isothiocyanate (BITC), and phenethyl isothiocyanate (PEITC). ITCs are bioactive compounds with anti-cancer and anti-bacterial properties, and have been shown to attenuate the effects of insulin resistance [19,20,21]. SFEN is formed via the hydrolysis of glucoraphanin, which is a glucosinolate present at high levels in radish [22]. SFEN has been reported to exhibit anti-mutagenic activity against food-derived mutagens [23] and anti-cancer activity in several cancer cell lines [24]. Many recent studies have reported that several ITCs, including ERC, SFN, AITC, and PEITC, exhibit strong anti-obesity effects, largely via anti-adipogenic activities [25,26,27,28]. However, the anti-adipogenic and anti-obesity effects of SFEN, and its underlying mechanisms of action, including which adipogenic transcription factors (e.g., C/EBPβ) are important, are still unclear.

Herein, we compared the inhibitory effects of several ITCs on adipogenesis and found that SFEN was the most effective inhibitor of adipocyte differentiation and lipid accumulation in 3T3-L1 adipocytes. When the present study was in progress, Chen et al. (2018) [28] also reported that SFEN exhibited anti-adipogenic effects. In addition to investigating anti-adipogenic effects, the present study also reports several novel findings. We determined that C/EBPβ is involved in the mechanism underlying the inhibitory effects of SFEN, as SFEN decreases C/EBPβ protein stability through post-translational modification. Furthermore, we found that the anti-adipogenic effect of SFEN was consistent in human adipose tissue-derived stem cells (ASCs); this result is reported here for the first time.

## 2. Materials and Methods

### 2.1. Reagents

Dulbecco’s modified Eagle’s medium (DMEM), MesenPRO RS medium, DMEM-Ham’s F12 Nutrient mixture (DMEM-F12), fetal bovine serum (FBS), bovine calf serum (BCS), and l-glutamin (GlutaMax) were purchased from Gibco (Grand Island, NY, USA). SFEN, SFN, and IBR were purchased from LKT Laboratories (St. Paul, MN, USA). ERC, BITC, PEITC, AITC, indomethacin, methylisobutylxanthine (IBMX), dexamethasone, insulin, Oil Red O powder, MG132, and the antibody against β-actin were purchased from Sigma-Aldrich (St. Louis, MO, USA). Antibodies against PPARγ and n-acetyl-leu-leu-norleucinal (ALLN) were obtained from Santa Cruz Biotechnology (Dallas, TX, USA). Antibodies against C/EBPα and C/EBPβ were purchased from Cell Signaling Biotechnology (Beverly, MA, USA). Isopropyl alcohol was obtained from Amresco LLC (Solon, OH, USA). The MACS, anti-CD31, CD45 microbeads and MACS separation buffer were purchased from Miltenyl Biotec (Bergisch Galdbach, Germany).

### 2.2. Cell Culture and Adipocyte Differentiation of 3T3-L1 Pre-adipocytes

For the cell culture, 3T3-L1 pre-adipocytes were purchased from ATCC (Manassas, VA, USA) and were maintained in DMEM supplemented with 10% BCS and 100 U/mL penicillin, 100 µg/mL streptomycin, and 0.25 µg/mL Fungizone^®^ (amphotericin B) under 10% CO_2_ and at 37 °C. To induce differentiation of the adipocytes, 3T3-L1 pre-adipocytes were seeded in 24-well plates at a density of 1.25 × 10^4^ cells per cm^2^, and then incubated until confluence was reached. The cells were incubated for two days in mouse adipocyte differentiation medium (MDM), which was DMEM supplemented with 10% FBS, 0.5 mM IBMX, 1 μM dexamethasone, 5 μg/mL insulin, 100 U/mL penicillin, 100 µg/mL streptomycin, and 0.25 µg/mL Fungizone^®^ (amphotericin B). After two days, the medium was replaced with DMEM containing 10% FBS and 5 μg/mL insulin. After incubation for a further two days, the cells were cultured in DMEM containing 10% FBS until the pre-adipocytes were fully differentiated, with the medium replaced every two days.

### 2.3. Isolation, Culture, and Adipocyte Differentiation of Human ASCs

Human ASCs were isolated from visceral adipose tissues (VATs) surrounding the intra-abdominal organs obtained from human donors (*n* = 5) undergoing gynecologic surgery. The clinical information of these patients is shown in Table 1. The procedure was approved by the Institutional Review Board of Seoul National University Hospital, South Korea (SNU-1003-009-311). We followed the provisions of the Declaration of Helsinki and obtained informed consent from the human donors for this study. Human ASCs were isolated as described previously [29]. Briefly, VAT was washed with sterile phosphate-buffered saline (PBS), and blood vessels were removed. The remaining tissue was dissociated with collagenase type IA (0.25 mg/mL PBS) for 1 h at 37 °C. The mixture of adipose tissue and collagenase was inverted gently every 10 min. After centrifugation at 500× *g* for 4 min, the stromal vascular fraction (SVF) was diluted with magnetic activated cell sorting system (MACS) buffer and filtered using a cell strainer. After centrifugation, the pellet was incubated with CD31 (endothelial cell marker) and CD45 (hematopoietic stem cell marker) micro beads for 15 min, and the negative selection of CD31 and CD45 was processed using the MACS. The mixture of cells with micro beads was centrifuged, and the pellet containing CD31- and CD45-SVFs was suspended with MesenPRO RS medium, specifically formulated for human ASCs, supplemented with 2 mM l-glutamine (GlutaMax), 100 U/mL penicillin, and 100 μg/mL streptomycin and plated in a cell culture dish and cultured in a 5% CO_2_ incubator. After three days, non-adherent cells were washed out using PBS. ASCs that adhered to the dishes were cultured in MesenPRO RS medium at least twice prior to experimental use. ASCs at passages 3–6 were used for experiments. To induce adipocyte differentiation, ASCs were seeded in 24-well plates at 0.75 × 10^4^ cells per cm^2^ and incubated in MesenPRO RS medium. After confluence, the cells were incubated with human adipocyte differentiation medium (HDM), which was DMEM-F12 supplemented with 10% FBS, 10 μg/mL insulin, 0.5 mM IBMX, 50 μM indomethacin, 1 μM dexamethasone, 100 U/mL penicillin, and 100 μg/mL streptomycin. The cells were left to differentiate for 14 days with the medium being changed every two days.

### 2.4. Oil Red O Staining

The mature adipocytes were fixed in 4% formalin for 20 min, and then washed with isopropyl alcohol. The fixed cells were then stained with Oil Red O solution for 15 min. Oil Red O solution was prepared by dissolving 0.25 mg of Oil Red O powder in 50 mL of 60% isopropyl alcohol, followed by filtering through a 0.45-μm membrane (Whatman, Maidstone, UK). After staining, the cells were washed twice with PBS. Thereafter, the Oil Red O stain was eluted with isopropyl alcohol, and the absorbance was measured at 515 nm (for 3T3-L1) and 495 nm (for human ASCs) using a spectrophotometer (Softmax pro 5; Molecular Devices, CA, USA).

### 2.5. Western Blot Assay

Western blot analysis was performed following the procedure described in our previous study [30]. Briefly, 3T3-L1 pre-adipocytes were seeded and cultured for two days in DMEM supplemented with 10% BCS. After confluence, the medium was changed to MDM with or without 5 or 10 μM SFEN. The protein concentration in each sample was determined using a dye-binding protein assay kit (Bio-Rad Laboratories, Hercules, CA, USA) according to the manufacturer’s instructions. Cell lysates underwent 10% sodium dodecyl sulfate-polyacrylamide gel electrophoresis and were transferred to a polyvinylidene difluoride membrane (GE Healthcare, Chicago, IL, USA). The membrane was blocked with 5% skim milk and incubated with a specific primary antibody followed by a horseradish peroxidase-conjugated secondary antibody. The protein bands were visualized using a chemiluminescence detection kit (Amersham Pharmacia Biotech, Little Chalfont, UK).

### 2.6. Quantitative Real Time (qRT) PCR

The 3T3-L1 pre-adipocytes were seeded onto 6-cm dishes at a density of 0.75 × 10^4^ cells per cm^2^ and cultured until confluence was reached. After confluence, cells were incubated in MDM with or without 5 or 10 μM SFEN. Total RNA was extracted using RNA-Bee reagent (Tel-Test, Friendswood, TX, USA) according to the manufacturer’s instructions. The amount of RNA was quantified using a NanoDrop ND-2000 spectrophotometer (Thermo Scientific, Waltham, MA, USA). Then, 1 μg of RNA was reverse-transcribed into complementary DNA (cDNA) using a cDNA synthesis kit (Takara Korea Biomedical, Seoul, South Korea). Diluted cDNA was added to a SYBR premixed Taq reaction mixture (BIORAD, CA, USA) containing 100 ng/mL of PCR primers. The sequences of the primers corresponding to adipogenic genes are as follows: PPARγ (forward, 5’-CGCTGATGCACTGCCTATGA-3’; reverse, 5’-AGAGGTCCACAGAGCTGATTCC-3’); C/EBPα (forward, 5’-CGCAAGAGCCGAGATAAAGC-3’; reverse, 5’-CACGGCTCAGCTGTTCCA-3’); C/EBPβ (forward, 5’-AGCGGCTGCAGAAGAAGGT-3’; reverse, 5’-GGCAGCTGCTTGAACAAGTTC-3’); β-actin (forward, 5’-TGTCCACCTTCCAGCAGATGT-3’; reverse, 5’-AGCTCAGTAACAGTCCGCCTAGA-3’) (Bioneer, South Korea). The qRT-PCR analysis was performed using a CFX Connect Real-Time PCR Detection system (Bio-Rad Laboratories). We calculated the relative mRNA expression according to delta CT method (2-ddCt) [31]. β-actin was used for normalization.

### 2.7. Statistical Analysis

The data are expressed as the means ± standard deviation. The data were analyzed by Student’s t-test for independent samples. *P*-values of < 0.05 or < 0.01 were taken to indicate statistical significance.

## 3. Results

### 3.1. SFEN Exhibits Stronger Anti-adipogenic Effects than Other ITCs

**Figure 1:**
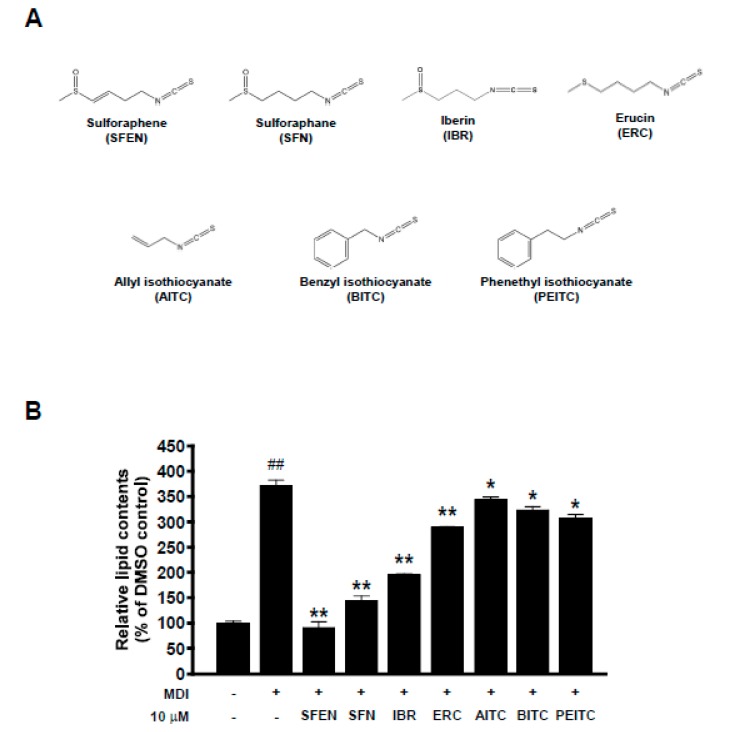
Comparison of effects of several isothiocyanates (ITCs) on differentiation medium-induced lipid accumulation in 3T3-L1 pre-adipocytes. (**A**) Structures of multiple ITCs. (**B**) Results of quantitative analysis using Oil Red O staining. To induce differentiation of the adipocytes, 3T3-L1 pre-adipocytes were seeded in 24-well plates at a density of 1.25 × 10^4^ cells per cm^2^, and were maintained in DMEM supplemented with 10% BCS under 10% CO^2^ and at 37 °C. Confluent cells were incubated for two days in mouse adipocyte differentiation medium (MDM) with treatment of each sample at a concentration of 10 μΜ. MDM contains DMEM supplemented with 10% FBS, 0.5 mM IBMX, 1 μM dexamethasone, 5 μg/mL insulin, 100 U/mL penicillin, 100 µg/mL streptomycin, and 0.25 µg/mL Fungizone^®^ (amphotericin B). After two days, the medium was replaced with DMEM containing 10% FBS and 5 μg/mL insulin with treatment of each sample. After incubation a further two days, the cells were cultured in DMEM containing 10% FBS with treatment of each sample. The medium for control was replaced every two days with DMEM containing 10% FBS. The mature adipocytes were fixed with 4% formalin and stained with Oil Red O on Day 6. The Oil Red O stain was extracted with isopropyl alcohol and the absorbance at 515nm was determined. We tested the effects of multiple ITCS with Oil Red O staining after inducing differentiation on six days by replacing each medium and treating sample at the same time every two days. Data are expressed as the means ± standard deviation (*n* = 3). ##, significant difference (*p* < 0.01) between the control and the MDM control. Significant differences between a treatment and the MDM control are indicated by * (*p* < 0.05) and ** (*p* < 0.01). SFEN, sulforaphene; SFN, sulforaphane; ERU, erucin; IBR, iberin; BITC, benzyl isothiocyanate; PEITC, phenethyl isothiocyanate; AITC, allyl isothiocyanate.

The structures of multiple ITCs with different side chains are shown in Figure 1A. We compared the ability of SFEN to inhibit adipogenesis to those of other ITCs, including SFN, IBR, ERC, AITC, BITC, and PEITC. The anti-adipogenic effects of these ITCs have been described previously [25,26,27,32]. To investigate whether SFEN exerts the strongest inhibitory effect against MDM-induced adipogenesis among this group of ITCs, we tested the effects of multiple ITCs at the same concentration (10 μΜ) in the presence of MDM, an adipogenic inducer. MDM treatment alone induced adipogenesis and increased the relative lipid content 3.7-fold to undifferentiated pre-adipocytes. SFEN reduced the lipid accumulation up to 75% compared to MDM-induced differentiated control in 3T3-L1 adipocytes, which was the strongest anti-adipogenic effect among the ITCs (Figure 1B).

### 3.2. SFEN Decreases MDM-Induced PPARγ and C/EBPα Protein and mRNA Expression in a Dose-Dependent Manner

**Figure 2:**
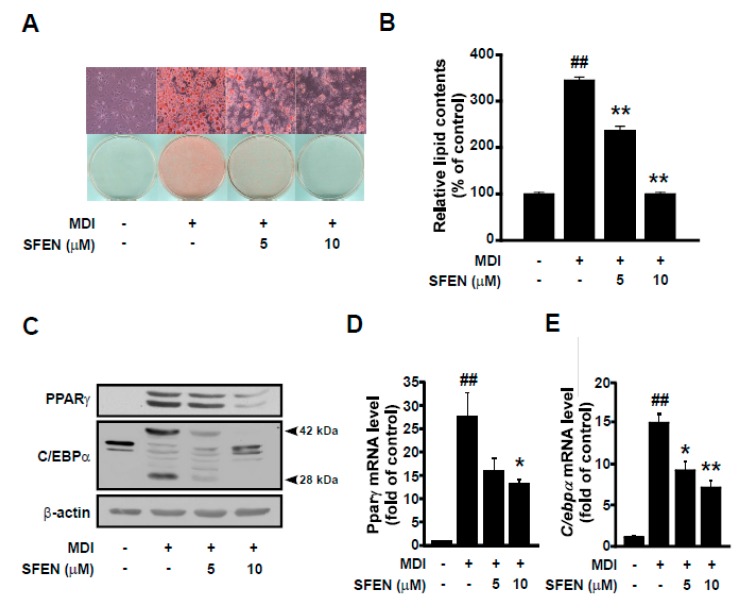
Effects of sulforaphene on differentiation medium-induced adipogenesis and the expression of two adipogenic genes—PPARγ and C/EBPα—in 3T3-L1 pre-adipocytes. (**A**) Image of differentiated 3T3-L1 adipocytes stained with Oil Red O (Magnification: 200×) (**B**) Quantification of intracellular lipid accumulation in differentiated 3T3-L1 adipocytes. (**C**) Western blot analysis of the protein expression levels of PPARγ (54, 57 kDa), C/EBPα (28, 42 kDa), and β-actin (43 kDa) as a housekeeping gene. Quantitative PCR analysis of the gene expression levels of (**D**) *Pparγ*, (**E**) *C/ebpα*, and *β-actin* as housekeeping gene. (**A**,**B**) 3T3-L1 pre-adipocytes were seeded at a density of 1.25 × 10^4^ cells per cm^2^ in 24-well plates and (**C**–**E**) 0.75 × 10^4^ cells per cm^2^ in 6 cm dishes. Confluent cells were incubated for two days in mouse adipocyte differentiation medium (MDM) with or without SFEN treatment at a concentration of 5 or 10 μΜ. After six days of differentiation, (**A**) the cell layer was stained with Oil Red O and (**B**) intracellular lipid accumulation was quantified. (**C**) The mature adipocytes were harvested by using 80 μL of RIPA buffer per dish for western blotting. 40 μg of protein was loaded per lane on 10% gel. (**D**, **E**) The mature adipocytes were harvested for qPCR and 1 μg of RNA was used for the synthesis of cDNA. Data were obtained from three independent experiments and are expressed as the means ± standard deviation (*n* = 3). ##, significant difference (*p* < 0.01) between the control and the MDM control. *, **, significant difference (*p* < 0.05, *p* < 0.01) between a treatment and the MDM control.

To investigate the effect of SFEN on adipogenesis, we carried out Oil Red O staining and examined lipid accumulation induced by MDM with or without SFEN treatment at a concentration of 5 or 10 μΜ. SFEN suppressed MDM-induced lipid accumulation effectively in a dose-dependent manner (Figure 2A). Quantitative analysis of Oil Red O staining revealed that MDM increased relative lipid content in differentiated cells by 3.45-fold compared to undifferentiated control cells. In cells treated with 10 μM SFEN, lipid accumulation was reduced to levels similar to those observed in the undifferentiated control cells (Figure 2B). To elucidate the mechanism underlying the inhibitory effects of SFEN on adipogenesis, we investigated the effects of SFEN on the expression levels of PPARγ and C/EBPα, which are master transcriptional regulators that modulate a cascade of transcriptional events in adipogenesis [9]. Treatment with SFEN effectively decreased the MDM-induced PPARγ and C/EBPα protein expression (Figure 2C) and mRNA expression (Figure 2D,E). These results indicate that SFEN inhibits the expression of the master adipogenic transcription factors PPARγ and C/EBPα at the RNA level. SFEN was not observed to be toxic at concentrations up to 10 μM (data not shown).

### 3.3. SFEN Exerts Anti-Adipogenic Effects at the Early Stage of Differentiation

**Figure 3:**
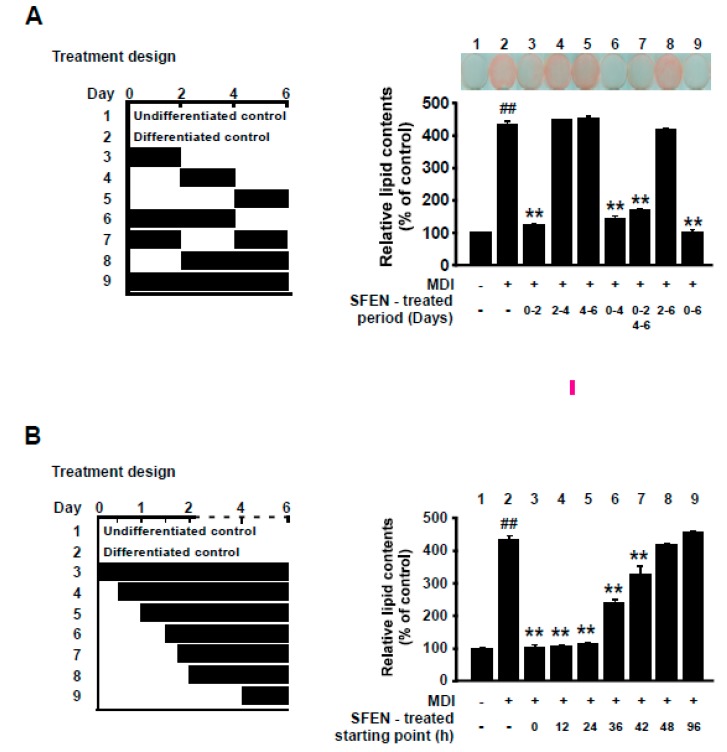
Effect of sulforaphene (SFEN) on differentiation medium-induced adipogenesis during the latter half of the early stage (24–48 h) of adipogenesis in 3T3-L1 pre-adipocytes. (**A**, left) The experimental design indicating the time points of each SFEN treatment after addition of mouse adipocyte differentiation medium (MDM). (**A**, right) Image of cells stained with Oil Red O, and intracellular lipid accumulation when cells were exposed to SFEN at different time points. (**B**, left) The experimental design indicating the time points of each SFEN treatment. (**B**, right) Results of quantitative analysis of Oil Red O-stained 3T3-L1 pre-adipocytes. We tested the effects of SFEN with Oil Red O staining after inducing differentiation for six days by replacing each medium and treating SFEN at the indicated time duration in “treatment design”. Data were obtained from three independent experiments and are expressed as the means ± standard deviation (*n* = 3). ##, significant difference (*p* < 0.01) between the control and the MDM control. **, significant difference (*p* < 0.01) between a treatment and the MDM control.

Growth-arrested pre-adipocytes go through three distinct stages to differentiate into mature adipocytes: the early stage (days 0–2), intermediate stage (days 3–4), and terminal stage (after day 4) [33,34,35]. Because SFEN significantly inhibited adipogenesis at 10 μM, we investigated the stage at which SFEN acts during adipogenesis. 3T3-L1 pre-adipocytes exposed to MDM were treated with 10 μM SFEN at various periods during the full process of adipocyte differentiation (Figure 3A, left). SFEN treatment significantly suppressed MDM-induced adipogenesis when 3T3-L1 cells were treated at the early stage (days 0–2) (Figure 3A, right). To further define the critical starting point when treated SFEN exerts inhibitory effects on adipocyte differentiation, we treated cells with 10 μM SFEN from different starting points between days 0 and 2 during differentiation (Figure 3B, left). When the cells were treated with SFEN from 12 h to 6 days and from 24 h to 6 days after MDM treatment, a complete reduction in lipid accumulation was observed in all treated cells after six days. However, when the cells were treated with SFEN from 36 h, 42 h, and 48 h after MDM treatment (SFEN absent between 0–36 h, 0–42 h, and 0–48 h after MDM treatment, respectively) the inhibitory effect of SFEN on lipid accumulation decreased in a time-dependent manner (Figure 3B, right). These results indicate that to inhibit adipogenesis, it is important for cells to be exposed to SFEN during a period including from 24 to 48 h after MDM treatment. The latter half time of the early stage of adipocyte differentiation indicates corresponding duration (24–48 h).

### 3.4. SFEN Reduces MDM-Induced Increases in C/EBPβ Protein Levels but Not mRNA Levels

**Figure 4:**
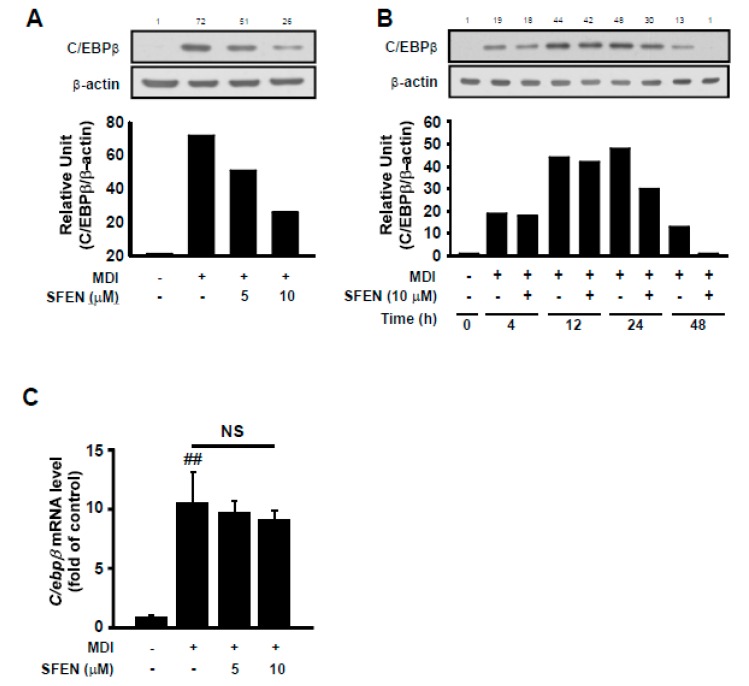
Effect of sulforaphene (SFEN) on differentiation medium-induced C/EBPβ expression in 3T3-L1 pre-adipocytes. (**A**) Western blot analysis indicated that SFEN attenuated the protein expression levels of C/EBPβ (35 kDa) and β-actin (43 kDa) as housekeeping gene in a dose-dependent and (**B**) time-dependent manner. (**C**) The gene expression levels of *C/ebpβ* and *β-actin* as housekeeping gene in 3T3-L1 pre-adipocytes were determined using real-time PCR. 3T3-L1 pre-adipocytes were seeded in 6 cm dishes at a density of 0.75 × 10^4^ cells per cm^2^. Confluent cells were incubated for two days in mouse adipocyte differentiation medium (MDM) with or without SFEN at a concentration of 5 or 10 μΜ. (**A**,**C**) At 48 h and (**B**) 4, 12, 24, and 48 h after the addition of MDM, (**A**,**B**) the cells were harvested by using 80 μL of RIPA buffer per dish to prepare samples for western blotting to determine the expression level of C/EBPβ. 40 μg of protein was loaded per lane on 10% gel. (**C**) The mature adipocytes were harvested for qPCR and 1 μg of RNA was used for the synthesis of cDNA. Data were obtained from three independent experiments. For Figure 4B, similar data were obtained from an independent repeated experiment. Data are expressed as the means ± standard deviation (*n* = 3). ##, significant difference between the control and the MDM control by (*p* < 0.01).

To elucidate the mechanism underlying the inhibitory effect of SFEN during the early stage of adipogenesis, we investigated the effect of SFEN on the expression of C/EBPβ protein, which is a key transcription factor that is mainly expressed and functions during the early stage for the transcriptional activation of PPARγ and C/EBPα. SFEN treatment effectively reduced MDM-induced C/EBPβ protein expression based on western blotting (Figure 4A,B). However, SFEN treatment had no significant effect on MDM-induced *C/ebpβ* mRNA expression (Figure 4C). These results indicate that the down-regulation of C/EBPβ is not due to reduced mRNA levels.

### 3.5. SFEN Induces Post-Translational Degradation of C/EBPβ by Decreasing Its Stability

**Figure 5:**
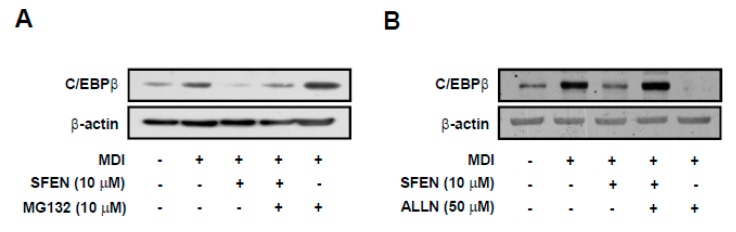
Effect of sulforaphene (SFEN) on the degradation of C/EBPβ in 3T3-L1 pre-adipocytes. (**A**) Western blot analysis of C/EBPβ protein (35 kDa) and β-actin (43 kDa) as a housekeeping gene expression levels after treatment with 10 μM MG132 (proteasome inhibitor) for 2 h and (**B**) after treatment with 50 μM ALLN (calpain inhibitor) for 1 h. 3T3-L1 pre-adipocytes were seeded in 6 cm dishes at a density of 0.75 × 10^4^ cells per cm^2^. Confluent cells were incubated for two days in mouse adipocyte differentiation medium (MDM) with or without 10 μΜ SFEN. (**A**) After two days, followed by treatment with 10 μM MG132 or (**B**) 50 μM ALLN, the cells were harvested by using 80 μL of RIPA buffer per dish to prepare samples for western blotting. 40 μg of protein was loaded per lane on 10% gel. The representative photographs obtained from three independent experiments are shown.

Because SFEN treatment did not alter MDM-induced *C/ebpβ* mRNA levels, we assessed the effect of SFEN on C/EBPβ protein stability. We sought to determine whether the decrease in C/EBPβ protein levels was due to the proteasomal-degradation pathway using the proteasome inhibitor MG132. SFEN treatment effectively reduced MDM-induced C/EBPβ protein levels (Figure 4A). However, co-treatment of SFEN with MG132 mitigated SFEN inhibition of MDM-induced increases in C/EBPβ protein levels (Figure 5A). We also investigated the calpain-dependent degradation pathway as an independent degradation pathway using the calpain inhibitor ALLN. Whereas treatment with SFEN alone effectively reduced MDM-induced C/EBPβ protein levels, co-treatment with SFEN and ALLN completely reversed the inhibitory effect of SFEN on MDM-induced C/EBPβ protein levels (Figure 5B). These results indicate that the down-regulation of C/EBPβ protein expression by SFEN is due to enhanced post-translational degradation via decreasing the stability of C/EBPβ.

### 3.6. SFEN Suppresses Adipogenesis in Human ASCs

**Figure 6:**
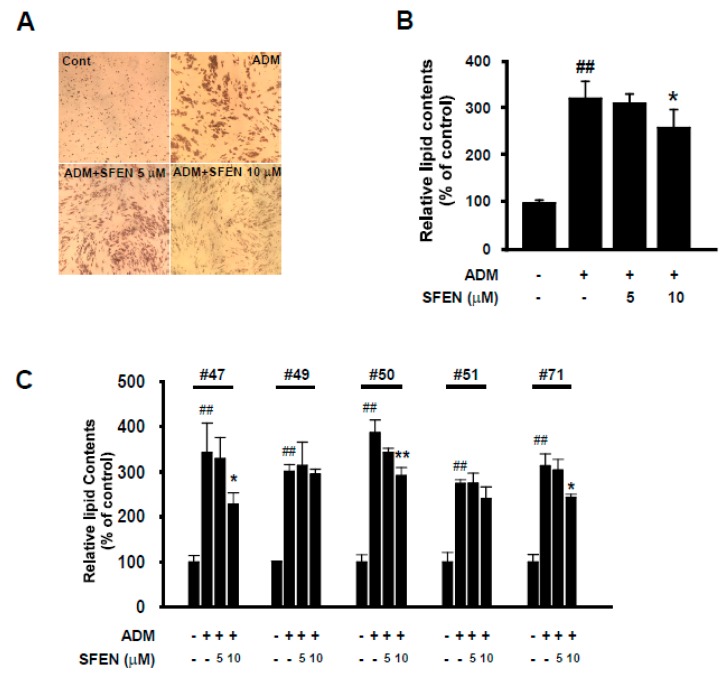
Effect of sulforaphene (SFEN) on adipocyte differentiation medium-induced adipogenesis in human adipose tissue-derived stem cells (ASCs). (**A**) Image of differentiated human ASCs from donor #50 stained with Oil Red O on day 14 after HDM treatment (Magnification: 200×). (**B**) Intracellular lipid accumulation in differentiated human ASCs. (**C**) Intracellular lipid accumulation in differentiated ASCs from individual donors. ASCs were seeded in 24-well plates at 0.75 × 10^4^ cells per cm^2^ and incubated in MesenPRO RS medium. Post-confluent ASCs were incubated with or without human adipocyte differentiation medium (HDM) for 14 days. HDM contains DMEM-F12 supplemented with 10% FBS, 0.5 mM IBMX, 50 μM indomethacin, 1 μM dexamethasone, 10 μg/mL insulin, 100 U/mL penicillin, and 100 μg/mL streptomycin. After 14 days, (**A**) the cells were then stained using Oil-Red O and (**B**,**C**) intracellular lipid accumulation was quantified. (**C**) Data were obtained from three independent experiments using three different passages of ASCs for each patient and (**B**) averaged five patients. Data are expressed as the means ± standard deviation (*n* = 3). ##, significant difference (*p* < 0.01) between the control and the HDM control. Significant differences between a treatment and the HDM control are indicated by * (*p* < 0.05) and ** (*p* < 0.01).

To investigate the anti-adipogenic effects of SFEN on human ASCs, in addition to the 3T3-L1 cell line, we isolated human ASCs from the VATs surrounding the intra-abdominal organs of five women (aged 45–71 years); the clinical information of these tissue donors is shown in Table 1. Unlike immortalized cell line 3T3-L1, human ASCs may show variety in cell populations and take longer to arrive at full differentiation (Day 14) compared to 3T3-L1 (Day 6) [36]. Thus, we discriminated HDM from MDM for efficient differentiation of ASCs. Instead of DMEM, we utilized DMEM-F12 which contains a wider variety of additional nutrients than DMEM and is the most commonly used medium to culture and to induce adipocyte differentiation of ASCs [37,38]. Additionally, in HDM 5 μg/mL more insulin was added in addition to 5 μg/mL insulin, 0.5 mM IBMX and 1 μM dexamethasone, the common three components for the induction of adipocyte differentiation. Furthermore, we additionally supplemented 50 μM indomethacin, which is reported to promote adipocyte differentiation of mesenchymal stem cells by upregulating PPARγ2 and C/EBPβ expression [36,39]. HDM treatment increased relative lipid accumulation by 3.2-fold in differentiated cells compared to undifferentiated controls. Consistent with the results observed in experiments with 3T3-L1 cells, SFEN treatment (10 μM) significantly reduced HDM-induced lipid accumulation in human ASCs (Figure 6A,B). However, the anti-adipogenic effect of SFEN varied according to various donors. SFEN exhibited anti-adipogenic effects in ASCs sampled from donors 47, 50, and 71, but not in ASCs sampled from donors 49 and 51 (Figure 6C). SFEN did not exhibit cytotoxicity in human ASCs based on the concentrations used in this experiment (data not shown).

## 4. Discussion

**Figure 7:**
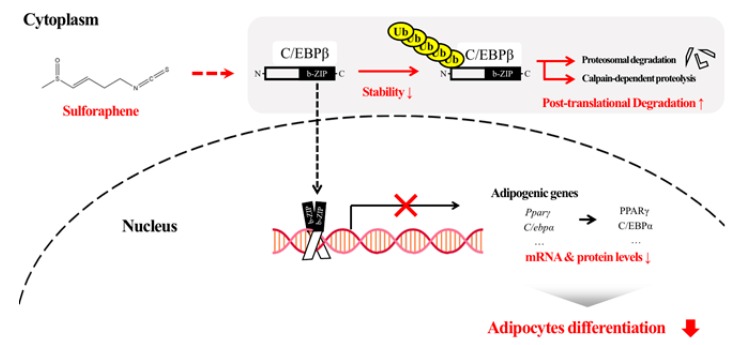
Diagram of the proposed mode of action of sulforaphene (SFEN) in adipogenesis. C/EBPβ is involved in the mechanism underlying the inhibitory effect of SFEN. SFEN decreases the levels of C/EBPβ through post-translational degradation via proteasome-dependent and calpain-dependent pathways.

In the present study, we found that SFEN exhibited a stronger anti-adipogenic effect in 3T3-L1 adipocytes compared to several other ITCs tested. This inhibitory effect of SFEN occurred at the early stage of adipogenesis (days 1–2) by decreasing the transcriptional expression of two master pro-adipogenic genes, *Pparγ* and *C/ebpα*. Treatment with SFEN led to a decrease in the stability of C/EBPβ, a major transcription factor of PPARγ and C/EBPα, which subsequently led to increased post-translational degradation. SFEN effectively destabilized C/EBPβ. This promoted protein degradation through proteasome-dependent and calpain-dependent pathways, and consequently led to decreased adipogenesis and lipid accumulation (Figure 7). This anti-adipogenic effect of SFEN was also observed in human ASCs, which could be a suggestion for further study as to whether SFEN can potentially regulate adipogenesis in humans or not.

The differentiation of 3T3-L1 pre-adipocytes by hormonal agents occurs via several transcriptional cascades [7]. C/EBPβ is a key transcription factor that activates the expression of PPARγ and C/EBPα, which are major regulators of adipogenesis. We showed the effect of SFEN on total lipid content in differentiated adipocytes with Oil Red O method in this study. There has been little study reported that C/EBPs or PPARγ regulate fatty acid desaturases or elongases, etc. However, interestingly, Xu et al. (2015) demonstrated that CEBP-2, a homolog of mammalian C/EBPs, modulates fatty acid desaturation by promoting the expression of fat-5 which is one of ∆9 desaturases converting saturated fatty acids to monounsaturated fatty acids in Caenorhabditis elegans [40]. Thus, we could eagerly speculate that SFEN modulated fatty acid desaturation, at least partially, in differentiated adipocytes via regulation of C/EBPs. However, in order to determine whether or not desaturation of fatty acids was actually altered by SFEN and, if so, exactly which fatty acids were mostly affected by SFEN, further experiments with gas chromatography analysis of intracellular fatty acids will be required.

C/EBPβ is rapidly expressed (4 h) after adipogenic induction, but lacks DNA-binding ability. After an extended period (~16 h), it is sequentially phosphorylated by MAPK and GSK3β [41]. This dual phosphorylation-induced conformational change in C/EBPβ can facilitate disulfide (S-S) bond formation and dimerization, finally activating its DNA-binding ability and allowing for transactivation capacity [42,43]. In its transcriptionally active form, C/EBPβ can form a homologous or heterologous dimer with other C/EBP proteins (e.g., C/EBP-α, -β, -δ, or -ε) via the basic leucine-rich zipper domain (b-zip) to stabilize itself and bind to DNA [44]. Otherwise, the inactive and unstable C/EBPβ protein monomer is degraded via the ubiquitin-proteasome pathway through the promotion of sumoylation [44,45], or nonfunctioning C/EBPs are rapidly cleared via calpain-dependent proteolysis [46,47,48]. We found that exposure to SFEN during the latter half of the early stage (24–48 h) of adipogenesis is crucial for the anti-adipogenic effect of SFEN to occur (Figure 3A,B). C/EBPβ protein levels were not altered by SFEN until 24 h after MDM treatment (Figure 4B). From these results, we hypothesize that SFEN affects the C/EBPβ post-translational modification process, including either phosphorylation or dimerization. We found that SFEN decreased the stability of C/EBPβ, which has been shown to cause C/EBPβ to enter the degradation pathway [44].

The mechanism underlying the post-translational degradation of C/EBPβ has not been extensively studied and remains controversial. However, several studies have suggested that C/EBPβ could be proteolyzed via an ubiquitin-proteasome- or calpain-dependent mechanism [44,46,47,49,50]. When b-zip, a leucine-rich domain of C/EBPβ, was truncated, C/EBPβ protein levels decreased in human melanoma A375 cells. The lower expression levels of truncated C/EBPβ were recovered via treatment with MG132, a proteasome inhibitor, which implied that the unstable C/EBPβ underwent proteasome-dependent degradation [44]. Nuclear levels of C/EBPβ protein have been reported to increase upon MG132 treatment in human intestinal Caco-2 cells [51]. Another study reported that the cleavage of C/EBPβ was inhibited by treatment with ALLN, a calpain inhibitor in the embryonic liver [48]. C/EBPβ protein levels reportedly increase upon treatment with various calpain inhibitors such as calpeptin and calpain inhibitor I and II in L6 and C2C12 skeletal muscle cells as well as in 3T3-L1 fibroblasts [46]. These results suggest that calpain protease is also involved in cleaving C/EBPβ. Our results revealed that the C/EBPβ expression levels suppressed by SFEN were rescued upon treatment with MG132 or ALLN, which indicates that SFEN treatment induces proteasome- or calpain-dependent degradation of C/EBPβ (Figure 5A,B). However, it seems that ALLN, in the absence of SFEN, rather decreased C/EBPβ expression in Figure 5B. Patel and Lane (2000) reported that ALLN (26 μΜ) inhibited adipogenesis in early stage by inhibition of p27 protein degradation [52]. Thus, it seems that ALLN could have opposite effects at the same time in regulation of C/EBPβ expression. First, ALLN could increase C/EBPβ expression by blocking protein degradation of C/EBPβ expression as a direct effect. Second, simultaneously, ALLN could inhibit adipogenesis by blocking protein degradation of p27 and, by doing so, resulting in reduction of C/EBPβ expression as an indirect effect in 3T3-L1 cells. Although we treated ALLN only for 1 h in the present study, we treated ALLN at higher concentration (50 μΜ) in the early stage of adipogenesis which is the same time point as the previous study of Patel and Lane (2000). It could be suggested that the inhibitory effect of ALLN by itself on adipogenesis through the regulation of p27 protein expression at least partially contributed to the reduction of C/EBPβ expression. Nevertheless, we could refer that, in the presence of SFEN, it enhanced ALLN-induced protein degradation of C/EBPβ from the 4th and 5th lane of Figure 5B.

Most health benefits of ITCs are attributed to the common N=C=S structure because it is electrophilic and highly reactive. ITCs can bind to certain proteins with nucleophilic amino acids containing thiol, amine, and hydroxyl groups, such as cysteine or lysine residues, thereby altering enzyme activity and signal transduction [53]. It has been suggested that C/EBP proteins are stabilized by forming dimers via b-zip, which allows the protein to avoid ubiquitin-proteasome-dependent degradation [44,54]. Kim et al. (2007) have reported that a cysteine residue at the C terminus or adjacent to b-zip is important for facilitating the dimerization of C/EBPβ through disulfide bond formation with sulfhydryl groups (-SH). Therefore, it is possible that the electrophilic carbon in ITCs can react with the cysteine residue in C/EBPβ required for dimerization. However, Oil Red O staining indicated that different ITCs inhibited MDM-induced adipogenesis in different ways (Figure 1B), even though they all had the N=C=S structure. Besides the N=C=S structure, the side chains of each ITC can also contribute to ITC bioactivity in terms of lipophilicity, side-chain length, molecular geometry, and chemical stability [27,55]. Given the results published in previous studies [56,57], our findings suggest that SFEN interacts with C/EBPβ at an earlier stage of adipogenesis than other ITCs (Figure 1B).

In this study, we found that SFEN exerts anti-adipogenic activity specifically at the early stage of differentiation, which is consistent with the results of Chen et al. [28], which showed that SFEN inhibited adipogenesis in 3T3-L1 adipocytes by activating the Hedgehog (Hh) signaling pathway. In addition to the confirmation of the anti-adipogenic activity of SFEN, our study further revealed several novel findings. First, we compared the inhibitory effects of several ITCs on adipogenesis and found that SFEN is the most effective inhibitor of adipogenesis in 3T3-L1 pre-adipocytes. Second, we elucidated that C/EBPβ plays a crucial role in the mechanism underlying the anti-adipogenic activity of SFEN. SFEN reduced the stability of C/EBPβ, resulting in the degradation of unstable C/EBPβ, and consequently leading to, the decreased expression of multiple adipogenic proteins such as PPARγ and C/EBPα, which are usually induced at the late stage of adipogenesis. It has been clearly shown that C/EBPβ and Hh signaling proteins can induce the expression of PPARγ and C/EBPα, respectively. It remains to be determined how or whether C/EBPβ interacts with the Hh signaling pathway, as suggested by Chen et al. [28]. Third, we investigated the anti-adipogenic effects of SFEN in both human ASCs and the 3T3-L1 cell line. Human adipose tissue contains a population of adipogenic progenitors such as ASCs, which have potential to be committed to become pre-adipocytes and differentiate to mature adipocytes, which process is called de novo adipogenesis [8,58,59]. We isolated human ASCs from the abdominal fat tissues of five women (aged 45–71 years). We found that on average, SFEN similarly exhibited significant anti-adipogenic effects on both murine pre-adipocytes (3T3-L1) and human adipose tissue-derived stem cells (ASCs), although it seems that the inhibitory effect of SFEN in human ASCs was weaker compared to that in 3T3-L1 cells at the same concentration (10 μM) due to cell type-specific effects.

The behavior of cells can be affected by various factors such as derived source (e.g., the type and location of tissues), species (e.g., Mus musculus, homo sapiens), and variances from donors. Thus, although we showed that the induction of adipogenesis by HDM treatment is significantly sufficient in ASCs from all five donors, anti-adipogenic effects of SFEN were variable. SFEN only exhibited anti-adipogenic effects in ACSs obtained from the adipose tissues of donors 47, 50, and 71 but not in those obtained from donors 49 and 51 (Figure 6C). The classification of obesity against donors can be made by WHR (waist/hip ratio) as well as by BMI described in Figure 6A [60,61]. When BMI was considered (#51 > #47 > #71 > #50 > #49), four donors whose BMI ≤ 25 could be classified as normal but donor #51 as obese (36.6 kg/m2). On the other hands, when WHR was considered (#47 > #71 = #50 > #49 > #51), four donors whose WHR were > 0.85 could be classified as obese whereas donor #51 (0.85) was classified as normal. Interestingly, when we considered age (#47 > #71 > #50 > #49 > #51), SFEN significantly inhibited adipogenesis in the oldest donor #47 (aged 71) while SFEN did not suppress adipogenesis in ASCs of the youngest donor #51 (aged 45). ASC responses could be influenced by donor characteristics such as age, sex, BMI, and WHR. However, since we examined using limited sample size, it is difficult to find clear correlation between age or BMI/WHR and adipogenesis. Future experiments are required investigating the effectiveness of SFEN treatment using ASCs samples from human subjects with various backgrounds on a larger scale. Furthermore, we observed the anti-obesity effect of SFEN in high fat diet (HFD)-induced mouse model (preliminary data by Lee at al. (unpublished results)). Tissue analysis of the in vivo model should be performed and anti-obesity effects of SFEN in a clinical study remain to be investigated.

## 5. Conclusions

In this study, we found that SFEN significantly inhibits adipogenesis and lipid accumulation in human ASCs and the 3T3-L1 cell line. This inhibitory effect was stronger than those of other ITC compounds found in cruciferous vegetables. We also found that SFEN decreased C/EBPβ stability, thereby lowering PPARγ and C/EBPα expression. Taken together, our study emphasizes the potential for SFEN to be used as a treatment for the prevention of obesity.

## Figures and Tables

**Table 1:** Clinical information of donors for adipose tissue-derived stem cells (ASCs).

Donor No.	Sex	WHR	BMI (kg/m^2^)	Age (Years Old)
#47	Female	0.98	24.4	71
#49	Female	0.91	21.6	50
#50	Female	0.94	23.0	54
#51	Female	0.85	36.6	45
#71	Female	0.94	23.7	58

Human visceral adipose tissues (VATs) samples were taken intra-abdominally from donors (*n* = 5) undergoing gynecologic surgery. WHR, waist to hip ratio; BMI, body mass index.

## Abbreviations

AITC	allyl isothiocyanate
ALLN	n-acetyl-leu-leu-norleucinal
ASC	adipose tissue-derived stem cells
BCS	bovine calf serum
BITC	benzyl isothiocyanate
b-zip	basic leucine zipper domain
C/EBPα	CCAAT/enhancer-binding protein α
C/EBPβ	CCAAT/enhancer-binding protein β
DMEM	Dulbecco’s modified Eagle’s medium
DMEM-F12	Dulbecco’s modified Eagle’s medium with Ham’s F12
ERU	erucin
FBS	fetal bovine serum
GSK3β	glycogen synthase kinase 3β
HDM	human adipocyte differentiation medium
Ibe	iberin
IBMX	3-isobutyl-1-methylxanthine
ITC	isothiocyanate
MACS	magnetic activated cell sorting system
MAPK	mitogen-activated protein kinase
MDM	mouse adipocyte differentiation medium
PBS	phosphate-buffered saline
PEITC	phenethyl isothiocyanate
PPARγ	peroxisome proliferator-activated receptor γ
SFEN	sulforaphene
SFN	sulforaphane
SVF	stromal vascular fraction
UCP	uncoupling protein
VAT	visceral adipose tissue
WHR	waist hip ratio
